# Comparative transcriptome analysis identifies genes associated with chlorophyll levels and reveals photosynthesis in green flesh of radish taproot

**DOI:** 10.1371/journal.pone.0252031

**Published:** 2021-05-27

**Authors:** Yuan-yuan Li, Min Han, Rui-hua Wang, Ming-gang Gao

**Affiliations:** Department of Bioengineering, Key Laboratory of Biochemistry and Molecular Biology in Universities of Shandong (Weifang University), Weifang University, Weifang, China; Youngstown State University, UNITED STATES

## Abstract

The flesh of the taproot of *Raphanus sativus* L. is rich in chlorophyll (Chl) throughout the developmental process, which is why the flesh is green. However, little is known about which genes are associated with Chl accumulation in this non-foliar, internal green tissue and whether the green flesh can perform photosynthesis. To determine these aspects, we measured the Chl content, examined Chl fluorescence, and carried out comparative transcriptome analyses of taproot flesh between green-fleshed “Cuishuai” and white-fleshed “Zhedachang” across five developmental stages. Numerous genes involved in the Chl metabolic pathway were identified. It was found that Chl accumulation in radish green flesh may be due to the low expression of Chl degradation genes and high expression of Chl biosynthesis genes, especially those associated with Part Ⅳ (from Protoporphyrin Ⅸ to Chl a). Bioinformatics analysis revealed that differentially expressed genes between “Cuishuai” and “Zhedachang” were significantly enriched in photosynthesis-related pathways, such as photosynthesis, antenna proteins, porphyrin and Chl metabolism, carbon fixation, and photorespiration. Twenty-five genes involved in the Calvin cycle were highly expressed in “Cuishuai”. These findings suggested that photosynthesis occurred in the radish green flesh, which was also supported by the results of Chl fluorescence. Our study provides transcriptome data on radish taproots and provides new information on the formation and function of radish green flesh.

## Introduction

Non-foliar plant organs such as the fruit of tomatoes, oranges, eggplants, and bananas, are commonly green in youth and lose chlorophyll (Chl) at maturity [1]. In contrast, the skin and flesh of the green radish is green throughout the developmental stages, remaining green even after harvesting for up to 6 months at low-temperature (-0.8 to -1°C) [2]. Therefore, the green-fleshed radish is an ideal subject for investigating the formation, retention, and function of the interior green part in non-foliar organs.

The green color of the plant is due to the presence of Chls, which are pigments essential for photosynthesis. The Chl metabolic pathway has been well studied in plant leaves and contains three different phases: Chl a biosynthesis, Chl cycle, and Chl degradation. Although Chl synthesis occurs in plastids, the enzymes involved in Chl a biosynthesis are encoded by nuclear genes in higher plants [3]. The Chl a synthesis pathway can be divided into four parts [4]. Part Ⅰ is the reaction of glutamate to 5-aminolevulinic acid (ALA, the precursor of Chls), with three enzymes (EARS, hemA, and hemL) involved in this process [5,6]. Part Ⅱ involves reactions from ALA to uroporphyrinogen Ⅲ, which are catalyzed by hemB, hemC, and hemD [7]. Part Ⅲ involves the reactions from uroporphyrinogen Ⅲ to protoporphyrin Ⅸ, with the aid of three enzymes–hemE, hemF, and hemY [7]. Part Ⅳ involves the reactions from protoporphyrin Ⅸ to Chl a. The first step in Part Ⅳ is the insertion of Mg^2+^ into protoporphyrin IX. This step is catalyzed by Mg-chelatase, which possesses three distinct subunits, chlD, chlI, and chlH [8]. In addition to Mg-chelatase, at least six other enzymes are also responsible for the formation of Chl a in part Ⅳ, including chlM, chlE, POR, DVR, chlG, and chlP [7]. The interconversion between Chl a and Chl b is known as the Chl cycle, which is the second phase of Chl metabolism. Five enzymes are involved in the Chl cycle, CAO, chlG, CLH, NOL (NYC1), and HCAR [9,10]. Chl degradation is the third phase of Chl metabolism, with SGR, CLH, PAO, PPD, and RCCR participating in this phase [11].

Excessive Chls or Chl intermediates can produce reactive oxygen species and harm biomolecules, while too few Chls will decrease the efficiency of photosynthesis. Thus, the Chl metabolism pathway is precisely regulated in plants, and the expression of genes involved in Chl metabolism may differ greatly depending on the organs, developmental stages, time, and environmental factors relating to each plant [12]. For example, Chl metabolic genes encoding hemA, chlH, chlE, and POR were found to be closely related to Chl content in the petals and leaves of chrysanthemum [13], while the gene encoding Mg-chelatase played an important role in the green petals of the carnation [12]. Compared with that of the golden variant of the fruit, the green kiwifruit exhibits a lower expression of *SGR2* during the maturation stages [1]. In tea leaves, Li et al. [14] found that the expression levels of *PORA* were significantly correlated with Chl a content, and the expression of two *NYC1* genes showed a significant correlation with Chl a and Chl b content. A recent study on the skin of “Xinlimei” radish taproot showed that several genes associated with Chl metabolism presented significant higher expressions during the taproot skin greening process, such as *hemA*, *chlH*, *POR*, *chlG*, *HCAR*, *CAO*, *NOL*, *CLH*, and *RCCR* [15].

Green plant tissues containing Chls are known to use solar energy for photosynthesis. To date, various strategies have been developed to measure and detect photosynthesis, among which Chl fluorescence is a popular method and is an *in vivo* photosynthesic probe [16]. Chl fluorescence is effective for monitoring photosynthesis occurring in plant internal tissues, where photosynthesis is difficult to measure accurately [15,17]. Furthermore, the genes involved in photosynthesis are often used as markers for plant photosynthesis, such as *RBCS*, *RBCL*, *PEPC*, and *LHCB* [1,18]. Although the photosynthetic performance of non-foliar organs and tissues has been widely assessed, limited research is available on the green flesh of radish taproots.

In this study, RNA sequencing (RNA-seq) was performed to compare gene expression profiles between the flesh of green-fleshed “Cuishuai” (GF) and white-fleshed “Zhedachang” (WF) radish varieties during taproot development. We comprehensively explored the differentially expressed genes (DEGs) in green and white flesh tissues during radish taproot development, targeting Chl metabolism and photosynthesis-related pathways. Chl contents, Chl fluorescence, and light response curves of the taproots of these two varieties were also measured. Our results form a sound basis for further studies on the formation, retention, and function of green flesh in radish taproots at the transcriptional level.

## Materials and methods

### Plant materials

The seeds of the radish cultivars GF and WF were sown in Weifang, Shandong, China on August 31, 2018. Taproots of GF and WF (stages 1 to 5; S1 Fig) were harvested every seven days from September 25 to October 23. In each stage, the flesh at the top of the taproot was separated and cut into approximately 0.5 cm^3^ pieces, which were then quick-frozen in liquid nitrogen and stored in a refrigerator at -80°C. In addition, at stage 5 (S5), the taproots of the two cultivars were pulled from the soil, immediately placed in ice, and taken back to the laboratory. These were then observed for Chl auto-fluorescence and light response curves were measured. Other white- and green-fleshed radish cultivars were planted in August 2020. When matured, radish flesh tissues were stored at -80°C following the steps described above for GF and WF. Each sample comprised three independent biological replicates.

### Chl analysis and Chl fluorescence observation and measurement

The Chl a and Chl b contents were measured using a spectrophotometer (Shimadzu, Kyoto, Japan) after extraction by soaking in 96% ethanol [19], and the total Chl content was calculated by adding the content of Chl a and Chl b. Freshly chopped pieces of GF or WF taproot were placed on a glass slide with a few drops of water, and covered a coverslip. Chl fluorescence was excited using a 488nm laser, and the signals were detected in the range of 639-701nm using a Zeiss LSM710 NLO confocal microscope (ZEISS, Berlin, Germany). The maximum quantum efficiency (FV/FM), effective quantum efficiency (ΦPSII), and electron transport rate (ETR) were measured using the IMAGING-PAM Chl Fluorometer (WALZ, Effeltrich, Germany) according to the method of Wittmann and Pfanz [17].

### RNA extraction, library construction and sequencing

Total RNA was isolated using the Trizol reagent (Promega, Madison, WI, USA), after which a Nano Photometer® spectrophotometer (IMPLEN, CA, USA) was used to check for the purity of the RNA. Next, RNA concentration was measured using the Qubit® RNA Assay Kit in a Qubit® 2.0 Fluorometer (Life Technologies, CA, USA), and RNA integrity was assessed using the RNA Nano 6000 Assay Kit of the Bioanalyzer 2100 system (Agilent Technologies, CA, USA). A total of 3 μg RNA per sample was used as the input material for the RNA sample preparations. Sequencing libraries were generated using the NEBNext® Ultra™ Directional RNA Library Prep Kit for Illumina® (NEB, Ipswich, MA, USA) according to manufacturer recommendations. Finally, 30 RNA libraries were constructed with the library quality assessed using the Agilent Bioanalyzer 2100 system. Each cDNA library was sequenced on an Illumina Hiseqxten-PE150 platform. Raw RNA-seq data have been deposited in the Sequence Read Archive (SRA) in NCBI. The BioProject number is PRJNA684971.

### Bioinformatics analysis

Raw RNA-seq reads were first filtered by removing adapter sequences, low-quality sequences (Sanger base quality≤20), and sequences with N content greater than 5%. The clean reads obtained were then mapped to the Radish reference genome (ftp://ftp.kazusa.or.jp/pub/radish) using HISAT2 software [20]. Subsequently, the featureCounts software was utilized to assign sequence reads to the genomic features [21]. DEGs between different samples were determined based on the DEGSeq R package [22] with a threshold of |log_2_FoldChange|≥2 and adjusted P-value≤0.01. The Benjamini and Hochberg [23] method was applied for the P-value adjustment.

All sequences were aligned to protein databases (UniProtKB/Swiss-Prot, NR, GO, KEGG) using BLASTX with an E-value≤10^−5^ to acquire their functional annotations. Gene Ontology (GO) analysis was performed using Goseq [24] based on three groups: molecular functions, biological processes, and cellular components. The GO terms with adjusted P-value≤0.05 were considered as significantly enriched terms. For Kyoto Encyclopedia of Genes and Genomes (KEGG)-based annotation analysis, the R/ClusterProfiler package [25] was used to test the statistical enrichment of DEGs of pathways with adjusted P-value≤0.05.

### Quantitative real-time PCR analysis

Gene-specific primers were designed using the Primer Premier 3.0 plus. The actin gene was used as an internal control to standardize the results. Primer pairs used in the qRT-PCR are shown in S1 Table. The qRT-PCR analysis was carried out using SuperRealPreMix Plus (SYBR Green) on a Bio-Rad iQ5 Real-Time PCR platform. The qRT-PCR reaction was initially performed at 95°C for 15 min, followed by 40 cycles of 10 s at 95°C and 30s at 60°C. Melting curves were used to verify primer specificity, and all reactions were performed in four replicates. The comparative Ct method (2^−ΔΔCT^) was used to calculate the relative expression levels of the target genes.

## Results

### Auto-fluorescence, photosynthetic activity, and Chl content in radish flesh

**Fig 1:**
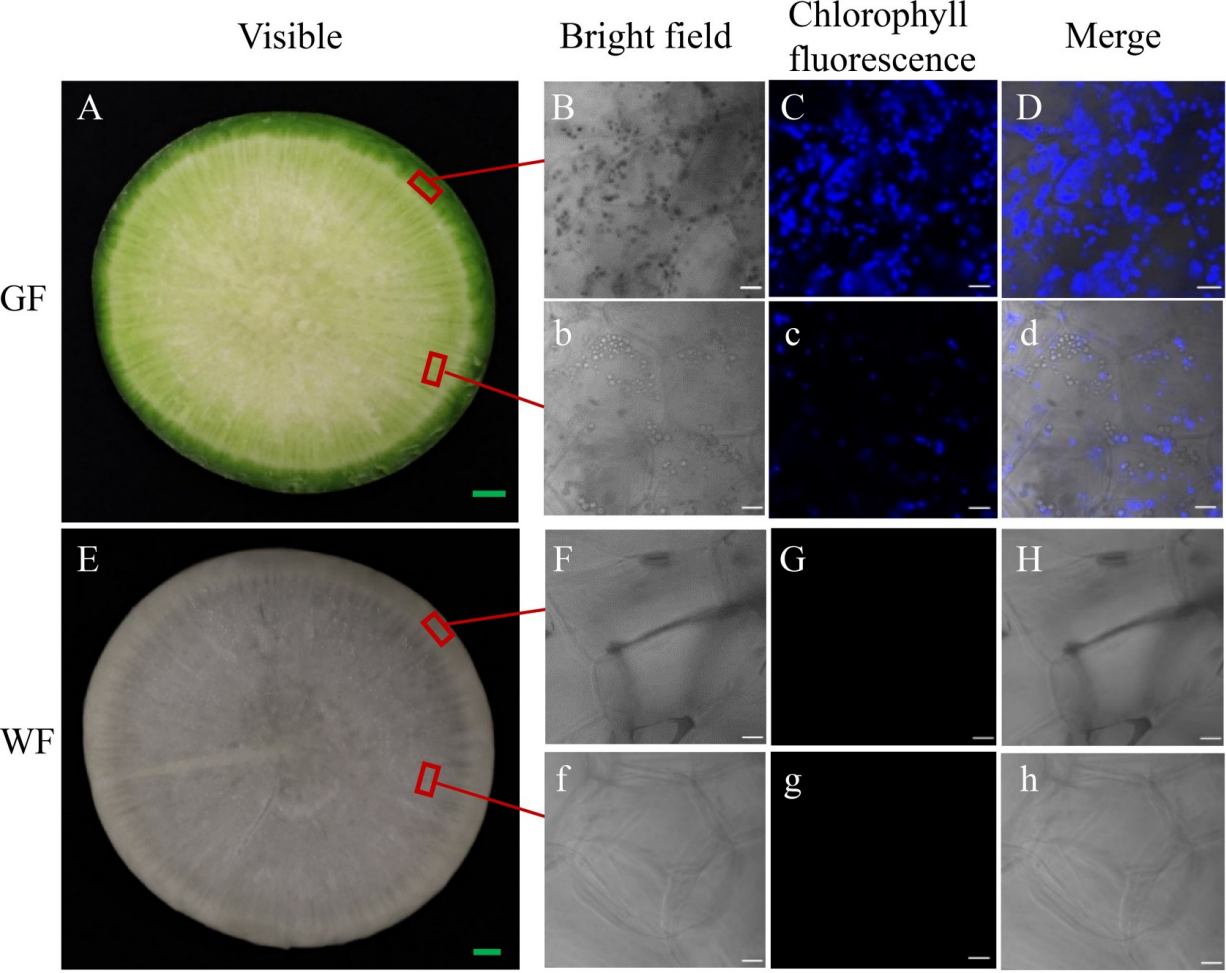
The skin and flesh phenotype of radish green flesh (GF) (A–D, b–d) and white flesh (WF) (E–H, f–h) at S5. **A, E,** cross section of GF (A) and WF (E) at visible light; **B, b, F, f,** micrograph at bright field; **C, c, G, g,** Chl auto-fluorescence image; **D, d, H, h**, the merged micrograph of bright field and Chl auto-fluorescence images. Green scale bars = 0.5cm, white scale bars = 20μm.

**Fig 2:**
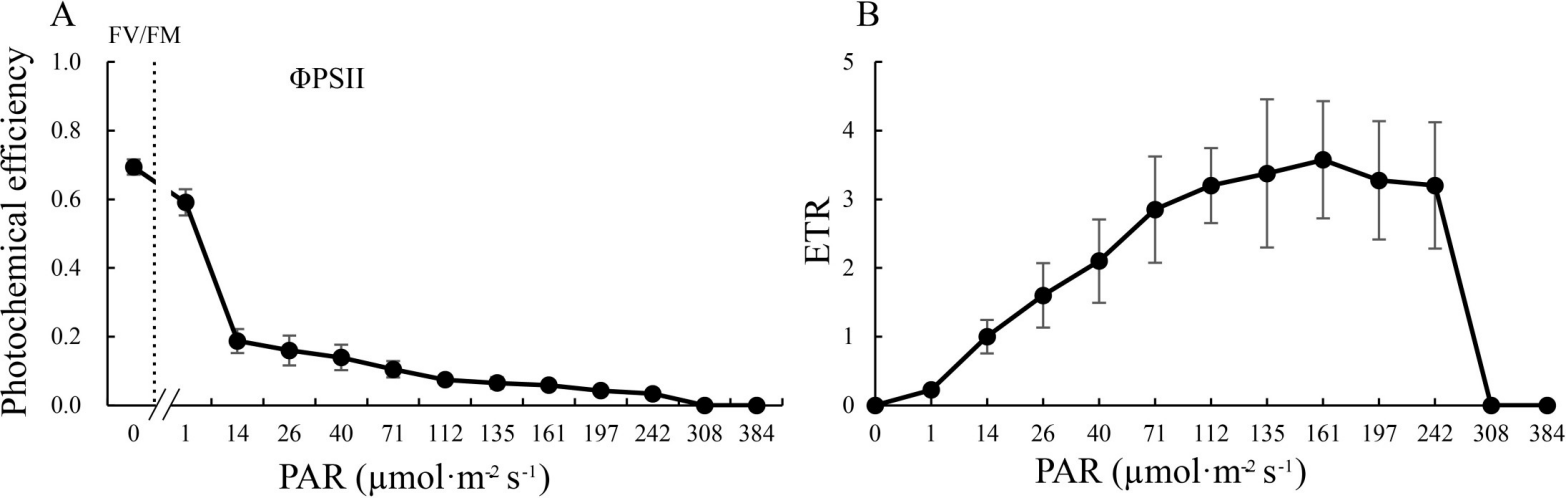
Light response of the PSII photochemical efficiency (A) and ETR (B) of GF tissues at S5. FV/FM, maximum quantum efficiency; ΦPSII, effective quantum efficiency; ETR, electron transport rate; PAR, photosynthetically active radiation.

GF is a green radish with dark green skin and green flesh (Fig 1A), while the WF has both white skin and flesh (Fig 1E). Chl auto-fluorescence was observed in the GF (Fig 1C), however, it was absent in the WF (Fig 1G and 1g). Furthermore, the FV/FM, ΦPSII and ETR of the GF were measured using the IMAGING-PAM. The FV/FM value was 0.694 for the GF (Fig 2A). With an increase in photosynthetically active radiation (PAR), ΦPSII significantly decreased and was completely suppressed at 308 μmol·m^-2^·s^-1^, while ETR increased and reached a maximum value of 3.575 at 161 μmol·m^-2^·s^-1^, before decreasing (Fig 2B).

**Fig 3:**
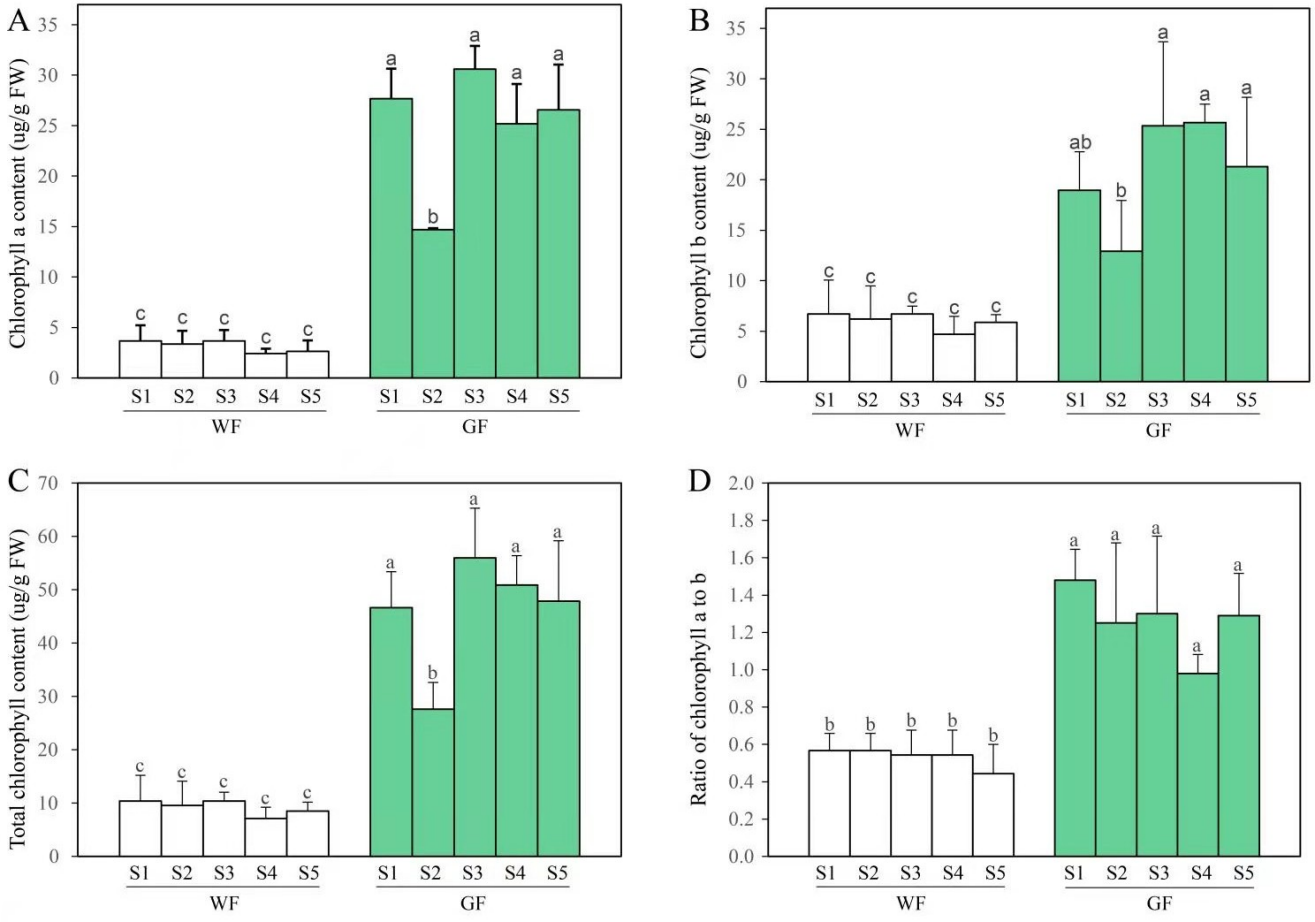
Chl content in flesh tissues at five developmental stages of WF and GF. **A**, Chl a content; **B**, Chl b content; **C**, Total Chl content; **D**, Ratio of Chl a to b. Each bar shows the mean values (±SD) of the three biological replicates, and different superscripted letters represents significant differences(P≤0.05).

The flesh tissues of GF and WF were collected from each of the five developmental stages (S1–S5), and Chl content was measured using the spectroscopic method. Only trace amounts of Chls were detected in the flesh tissues of WF, whereas numerous Chls were identified in the GF across all five stages of development (Fig 3). In the GF, Chl a, Chl b and total Chl content remained at a high level across all stages. In the WF, no significant differences were observed for Chl a, Chl b, and total Chl content across the five stages. It is worth noting that Chl a/b ratio did not change significantly during the developmental period. It was 0.98–1.48 in GF and 0.44–0.57 in WF.

### RNA-seq analysis

Radish flesh tissues of five corresponding stages from GF and WF were used to construct 30 independent libraries, which were then sequenced using the Illumina Hiseqx-ten-PE150 platform. The sequencing results are presented in Table 1. A total of 342.86 M and 337.83 M clean reads were generated from the GF and WF libraries, respectively. A total of 204.22 G clean bases were obtained, with an average of 6.81 G clean bases per library. The uniquely mapped reads ranged from 13.54–20.25 M with a mean of 15.81 M per library. Overall, approximately 80.53% (79.42–81.77% in GF) and 80.55% (79.29–81.28% in WF) of clean reads were aligned to the reference radish genome (http://radish.kazusa.or.jp/).

**Table 1:** Summary of thirty libraries.

Sample	Raw reads	Clean reads	Clean bases (G)	Q30 (%)	GC content (%)	Uniquely mapped reads	Percentage of total mapped reads (%)
GFS1-1	21,631,414	21,131,460	6.34	90.41	46.66	15,165,912	81.77
GFS1-2	21,559,420	21,175,552	6.35	88.53	46.86	14,896,168	80.84
GFS1-3	21,658,843	21,212,655	6.36	90.60	46.12	15,135,050	81.29
GFS2-1	25,126,450	24,625,141	7.39	89.88	46.34	17,324,388	80.73
GFS2-2	23,783,993	23,361,222	7.01	90.00	46.34	16,530,299	80.92
GFS2-3	25,140,994	24,268,951	7.28	90.59	46.23	17,324,942	81.11
GFS3-1	23,301,043	22,810,394	6.84	90.13	46.67	15,929,123	79.73
GFS3-2	21,322,093	21,006,993	6.30	90.02	46.88	14,787,564	80.67
GFS3-3	23,533,656	22,769,017	6.83	90.44	46.25	15,848,088	79.42
GFS4-1	29,065,936	28,496,744	8.55	90.36	46.63	20,246,541	81.29
GFS4-2	24,098,235	23,628,036	7.09	88.78	46.49	16,349,139	79.59
GFS4-3	20,268,744	19,743,435	5.92	89.06	46.53	13,907,130	80.20
GFS5-1	21,481,146	21,035,806	6.31	87.78	46.79	14,676,998	80.14
GFS5-2	24,461,033	23,989,156	7.20	88.52	46.89	16,740,508	80.17
GFS5-3	23,970,528	23,609,581	7.08	88.46	46.68	16,437,092	80.10
WFS1-1	22,040,591	21,373,563	6.41	91.45	47.06	14,959,809	81.15
WFS1-2	28,915,323	28,430,551	8.53	90.06	46.97	18,927,250	79.29
WFS1-3	26,034,988	25,348,252	7.60	91.10	46.99	17,781,282	80.78
WFS2-1	21,813,316	21,418,914	6.43	91.35	47.10	14,986,125	81.28
WFS2-2	21,715,480	21,227,940	6.37	90.29	46.90	14,237,963	79.43
WFS2-3	23,777,376	23,351,612	7.01	90.97	47.01	16,043,466	80.60
WFS3-1	23,905,519	23,484,048	7.05	90.65	47.25	15,993,994	79.93
WFS3-2	22,036,315	21,590,949	6.48	90.53	47.23	14,656,457	79.78
WFS3-3	20,169,800	19,764,169	5.93	90.77	47.20	13,536,532	80.12
WFS4-1	20,034,998	19,602,736	5.88	87.79	47.20	13,752,166	81.06
WFS4-2	23,870,082	23,359,836	7.01	90.64	47.07	16,151,091	80.63
WFS4-3	23,557,136	22,854,742	6.86	91.56	46.87	16,077,906	81.19
WFS5-1	22,793,567	22,451,363	6.74	91.03	47.09	15,516,108	80.79
WFS5-2	23,259,669	22,734,362	6.82	91.41	47.05	15,867,251	81.11
WFS5-3	21,234,034	20,834,848	6.25	91.31	47.07	14,564,077	81.08

### Validation of the RNA-seq data by qRT-PCR

**Fig 4:**
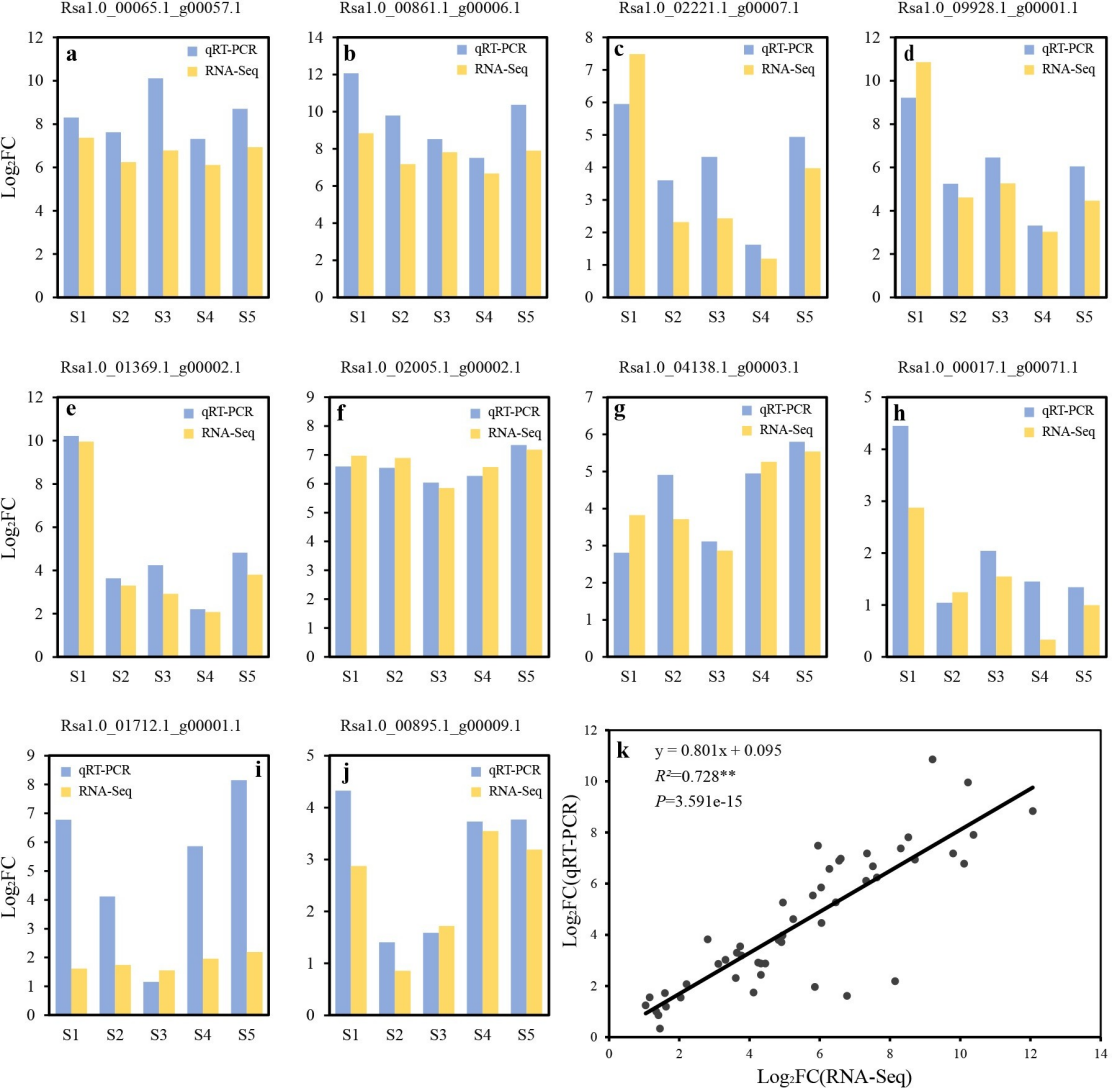
Validation of DEGs by qRT-PCR. **a–j**, Comparison of the Log_2_FC values of 10 different expressed genes at five development stages between qRT-PCR and RNA-Seq; **k**, Coefficient analysis of the log_2_FC between data of RNA-Seq (x-axis) and qRT-PCR (y-axis). **indicates a significant difference at level p≤0.01.

Ten DEGs were randomly selected to confirm the RNA-seq results using qRT-PCR. The expression patterns of selected genes were similar to the results of RNA-Seq (Fig 4A–4J) and a significant correlation was found between them (R^2^ = 0.728, P = 3.591e-15) (Fig 4K). This showed that the results of RNA-seq analysis were consistent with those of qRT-PCR analysis. The qRT-PCR and RNA-seq values for the target genes are listed in S2 Table.

### DEGs analysis

**Fig 5:**
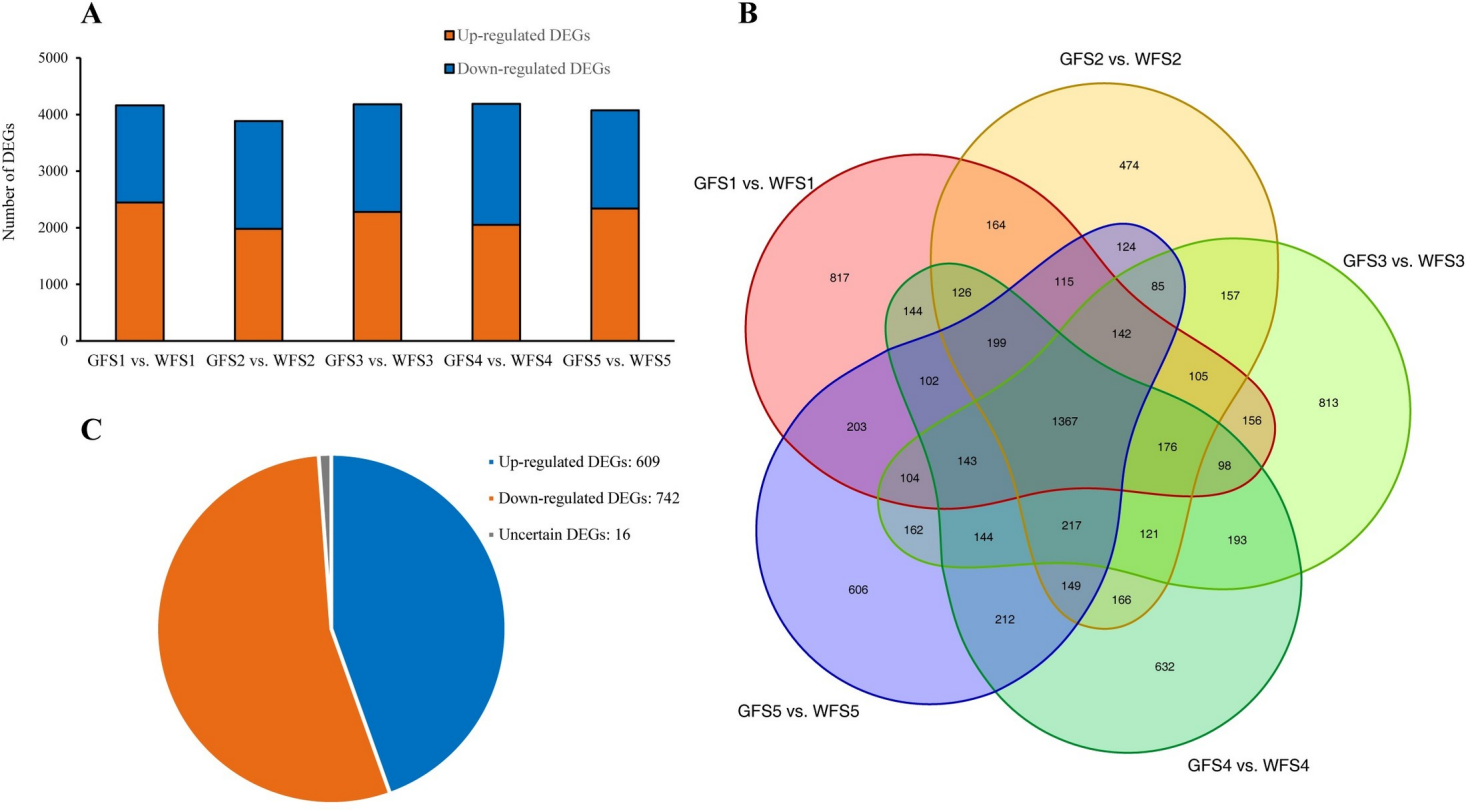
Distribution of DEGs obtained from the taproot flesh transcriptomes of GF and WF. **A**, DEGs between the GF and WF at five corresponding stages; **B**, Venn diagram showing the overlap in DEGs between GF and WF at the five developmental stages. The number in the cycle indicating the number of DEGs; **C**, Distribution of 1367 DEGs, which were consistently differentially expressed across the five stages.

To obtain the DEGs between GF and WF, the GF was compared to the WF at the corresponding stage (GFS1 vs. WFS1, GFS2 vs. WFS2, GFS3 vs. WFS3, GFS4 vs. WFS4, and GFS5 vs. WFS5). In summary, 8416 DEGs between GF and WF were detected at five stages (S3 Table, Fig 5). A total of 4161, 3887, 4183, 4189 and 4074 DEGs were obtained for S1, S2, S3, S4 and S5, respectively (Fig 5A). It should be noted that a total of 1367 DEGs were differentially expressed across the five stages (Fig 5B), of which 609 genes were up-regulated and 742 genes were down-regulated (Fig 5C).

**Fig 6:**
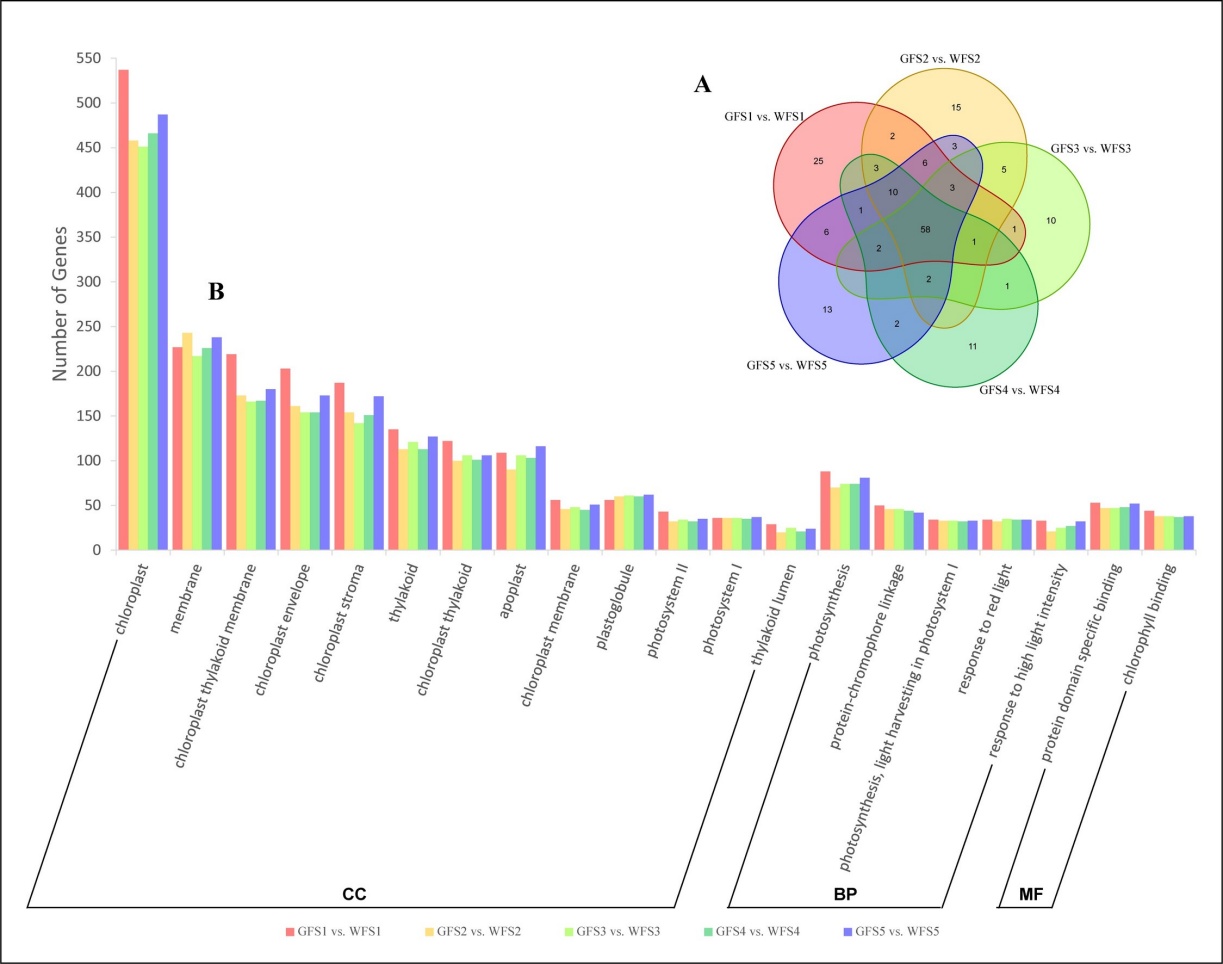
Gene Ontology (GO) enrichment analysis of annotated DEGs between the GF and WF across five developmental stages. **A**, Venn chart showing the overlap in GO enrichment terms at five stages. The number in the cycle indicating the number of terms. **B**, Twenty common GO terms enriched at all five stages. CC, cellular components; BP, biological process; MF, molecular function.

We performed the GO functional enrichment analysis using GOseq software to further understand the functions of these DEGs. To summarize, 180 enriched GO terms were identified across the five stages (Fig 6 and S4 Table). At S1, the number of enriched GO terms was 118, which was the highest across five stages. A total of 108 GO terms were identified at S2. At S3, only 83 GO terms were identified. At S5 and S6, 91 and 106 were identified, respectively. More than 32% of the GO terms [58] were simultaneously enriched across all five stages (Fig 6A). Photosynthetic-related terms, such as photosynthesis, photosystem I, photosystem II, light harvesting in photosystem I, chloroplast, chloroplast membrane, and chloroplast thylakoid, were included in these 58 terms (Fig 6B). The GO terms of 609 constantly up-regulated and 742 persistently down-regulated DEGs across the five stages were also analyzed. With regard to DEGs constantly up-regulated in GF compared with WF, chloroplast, chloroplast thylakoid membrane, chloroplast envelope, thylakoid, chloroplast thylakoid, photosynthesis and chloroplast stroma were the seven most enriched GO terms; and chloroplast, chloroplast stroma, chloroplast envelope, chloroplast thylakoid membrane, protein-chromophore linkage, response to red light and response to far-red light were the seven most enriched GO terms among DEGs persistently down-regulated in GF vs. WF (S2 Fig). The results of GO enrichment showed that many DEGs could function in the chloroplast or on the chloroplast envelope and may play important roles in the photosynthetic process.

Additionally, we performed KEGG pathway enrichment analysis of these DEGs. In summary, 273 DEGs were significantly enriched in ten KEGG pathways among the different stages (P value≤0.05) (S5 and S6 Tables). There were five pathways enriched at all five stages, including photosynthesis (ko00195), photosynthesis-antenna proteins (ko00196), glyoxylate and dicarboxylate metabolism (ko00630), carbon fixation in photosynthetic organisms (ko00710), and porphyrin and Chl metabolism (ko00860). The results of KEGG pathway enrichment analysis were similar to those of the GO enrichment.

### Chl metabolic pathway

**Fig 7:**
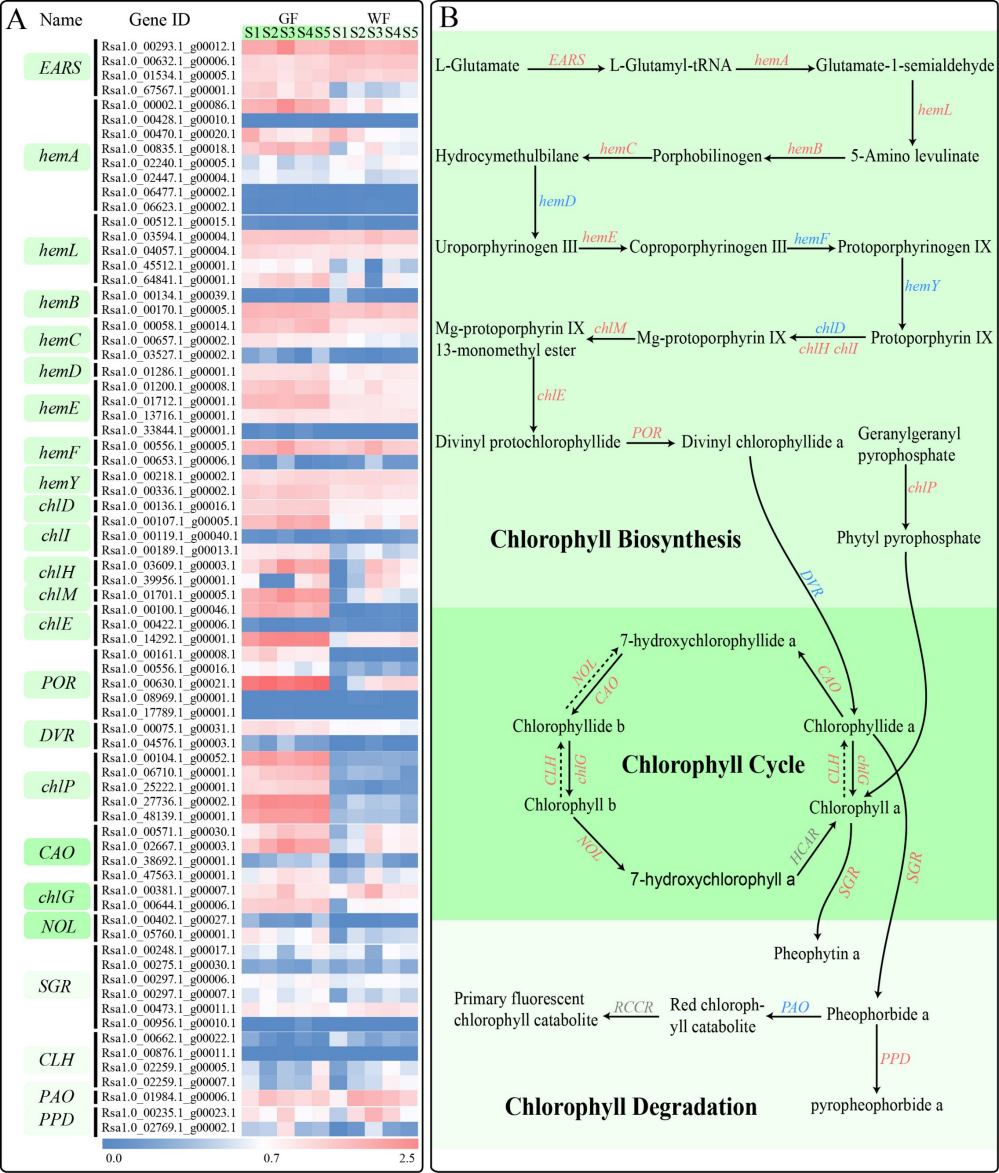
Chl metabolic pathway and expressions of Chl metabolic genes. **A**, A heatmap of the expressions of Chl metabolic genes in GF and WF radish taproot flesh at five developmental stages. **B**, The pathway of Chl metabolism. EARS, glutamyl-tRNA synthetase; hemA, glutamyl-tRNA reductase; hemL, glutamate-1-semialdehyde 2,1-aminomutase; hemB, por-phobilinogen synthase; hemC, hydroxymethylbilane synthase; hemD, uroporphyrinogen-III synthase; hemE, uroporphyrinogen decarboxylase; hemF, coproporphyrinogen III oxidase; hemY, protoporphyrinogen/coproporphyrinogen III oxidase; chlD, Mg-chelatase subunit D; chlI, Mg-chelatase subunit I; chlH, Mg-chelatase subunit H; chlM, magnesium-protoporphyrin O-methyltransferase; chlE, magnesium-protoporphyrin IX monomethyl ester (oxidative) cyclase; POR, protochlorophyllide reductase; DVR, divinyl chlorophyllide a 8-vinyl-reductase; chlG, Chl a synthase; chlP, geranylgeranyl diphosphate a reductase; CAO, chlorophyllide a oxygenase; NOL, chlorophyll(ide) b reductase; HCAR, 7-hydroxymethyl Chl a reductase; SGR, magnesium dechelatase; CLH, chlorophyllase; PAO, pheophorbide a oxygenase; PPD, pheophorbidase; RCCR, red chlorophyll catabolite reductase. Genes showing significant differential expression in at least one stage are indicated in red; Genes showing no significant differential expression are indicated in blue; Genes that were not detected in this study are indicated in grey.

Many genes that participate in the Chl metabolic pathway have already been identified and characterized in higher plants. We identified a total of 74 genes involved in this pathway in the present study, among which 34 genes showed significantly differential expression levels between GF and WF (Fig 7). The transcription levels of 12 genes were continuously higher in GF than those in WF from S1 to S5, including five *chlP* genes (Rsa1.0_00104.1_g00052.1, Rsa1.0_06710.1_g00001.1, Rsa1.0_25222.1_g00001.1, Rsa1.0_27736.1_g00002.1, and Rsa1.0_48139.1_g00001.1), two *chlE* genes (Rsa1.0_00100.1_g00046.1 and Rsa1.0_14292.1_g00001.1), two *POR* genes (Rsa1.0_00161.1_g00008.1 and Rsa1.0_00630.1_g00021.1), one *chlI* gene (Rsa1.0_00107.1_g00005.1), one *chlM* gene (Rsa1.0_01701.1_g00005.1), and one *CAO* gene (Rsa1.0_02667.1_g00003.1). The expression levels of four genes in GF were significantly higher than those in WF tissues at four of the five developmental stages, including *EARS* (Rsa1.0_67567.1_g00001.1), *hemA* (Rsa1.0_00835.1_g00018.1 and Rsa1.0_00002.1_g00086.1), and *POR* (Rsa1.0_00556.1_g00016.1). Three genes, *hemL* (Rsa1.0_45512.1_g00001.1), *chlH* (Rsa1.0_03609.1_g00003.1) and *chlI* (Rsa1.0_00189.1_g00013.1) were more highly expressed in GF than those in WF tissues at three different stages. Compared to the WF, *hemL* (Rsa1.0_64841.1_g00001.1), *chlG* (Rsa1.0_00644.1_g00006.1), *CAO* (Rsa1.0_00571.1_g00030.1), *NOL* (Rsa1.0_00402.1_g00027.1) and *hemC* (Rsa1.0_03527.1_g00002.1), showed significantly up-regulated expression in GF at the two stages. Five genes had notably higher expression levels in GF than those in the WF at only one stage: *NOL* (Rsa1.0_05760.1_g00001.1), *SGR* (Rsa1.0_00297.1_g00007.1) and *PPD* (Rsa1.0_00235.1_g00023.1) at S1, *PPD* (Rsa1.0_02769.1_g00002.1) at S2, and *hemE* (Rsa1.0_01712.1_g00001.1) at S5. The transcriptional levels of three genes, comprising *chlH* (Rsa1.0_39956.1_g00001.1) and *CLH* (Rsa1.0_02259.1_g00005.1 and Rsa1.0_02259.1_g00007.1), were higher at the early stage, but were lower at later stages in GF than those in WF tissues. Two genes, *hemB* (Rsa1.0_00134.1_g00039.1) and *CLH* (Rsa1.0_00662.1_g00022.1) had significantly lower expression levels in GF tissues than those in WF tissues.

Pearson’s correlation analysis was performed to analyze the correlation between the expression levels of 34 DEGs that participated in Chl metabolism and the Chl content for each stage. The expression levels of 28 (82.35%), 27 (79.41%), 28 (82.35%), and 27 (79.41%) DEGs were significantly correlated with the contents of Chl a, Chl b, total Chl and Chl a/b, respectively (P≤0.01, S3 Fig). The 12 DEGs with continuously high expression levels in GF from S1 to S5 were all significantly positively correlated with the Chl content. These genes may play important roles resulting in the differences in Chl content between GF and WF.

### Carbon fixation pathway

**Fig 8:**
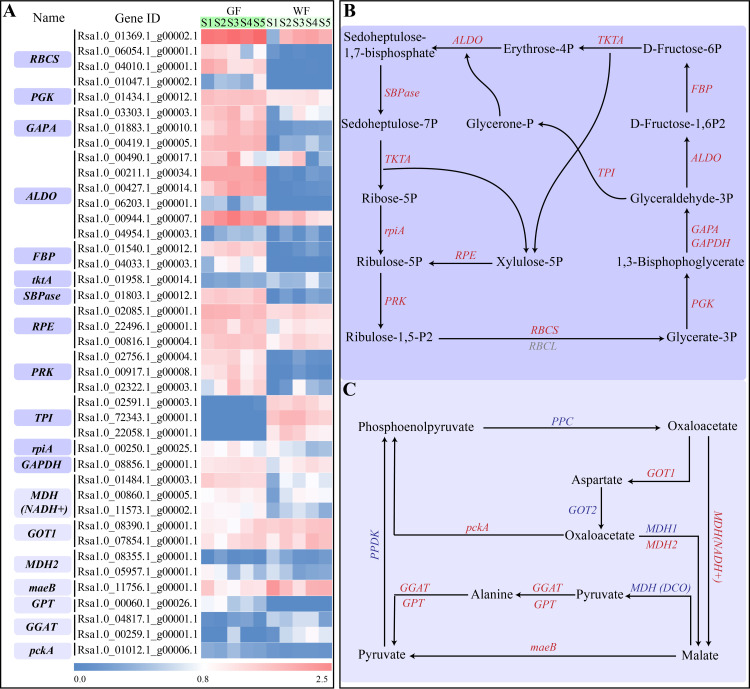
Carbon fixation pathway and expressions of genes involved in carbon fixation. **A**, A heatmap of the expressions of carbon fixation DEGs in GF and WF radish taproot flesh at five developmental stages. **B**, The Calvin cycle. **C**, The C_4_-Dicarboxylic acid cycle. RBCS, ribulose-bisphosphate carboxylase small chain; RBCL, ribulose-bisphosphate carboxylase large chain; PGK, phosphoglycerate kinase; GAPA, glyceraldehyde-3-phosphate dehydrogenase; ALDO, fructose-bisphosphate aldolase; FBP, fructose-1,6-bisphosphatase I; tktA, transketolase; SBPase, sedoheptu-lose-bisphosphatase; RPE, ribulose-phosphate 3-epimerase; PRK, phosphoribulokinase; TPI, triosephosphate isomerase; GAPDH, glyceraldehyde 3-phosphate dehydrogenase; rpiA, ribose 5-phosphate isomerase A; MDH1, malate dehydro-genase 1; MDH (NADH+), malate dehydrogenase (NADH+); GOT1, aspartate aminotransferase; MDH2, malate dehy-drogenase 2; maeB, malate dehydrogenase; GPT, alanine transaminase; GGAT, glutamate-glyoxylate aminotransferase; pckA, phosphoenolpyruvate carboxykinase (ATP); ppDK, pyruvate, orthophosphate dikinase; PEPC, phosphoenolpy-ruvate carboxylase; GOT2, aspartate aminotransferase, mitochondrial; MDH (DCO), malate dehydrogenase (decarboxylating). Genes showing significant differential expression in at least one stage are indicated in red; Genes showing no significant differential expression are indicated in blue; Genes that were not detected in this study are indicated in grey.

A total of 114 genes participating in the carbon fixation pathway were identified in this study, which included 41 DEGs (Fig 8 and S7 Table). Carbon reactions of photosynthesis, including the C_3_ carbon cycle, C_4_ carbon cycle and crassulacean acid metabolism, convert CO_2_ into carbohydrates. The C_3_ carbon or Calvin cycle is the most essential pathway for CO_2_ assimilation. We found that 29 DEGs encoding 12 key enzymes were enriched in the C_3_ carbon cycle. Among them, there were 25 significantly up-regulated genes, including the orthologs of *RBCS*, *PGK*, *GAPA*, *ALDO*, *FBP*, *PRK* and *RPE*. In addition, 12 DEGs were enriched in the C_4_ carbon cycle. Of these, six genes had higher expression levels in the GF than those in the WF at least one of the five stages. Three *PPDK* genes and six *PEPC* genes (key genes of the C_4_ pathway) were also identified in our transcriptome data. All *PPDK* genes and two *PEPC* genes were expressed at extremely low levels in the GF. Although another four *PEPC* genes were expressed at the middle levels, no difference was detected in their expression levels between GF and WF tissues. Therefore, it is unlikely that the C_4_ pathway exists in the radish green flesh.

### Photosynthesis-related Pathways

**Fig 9:**
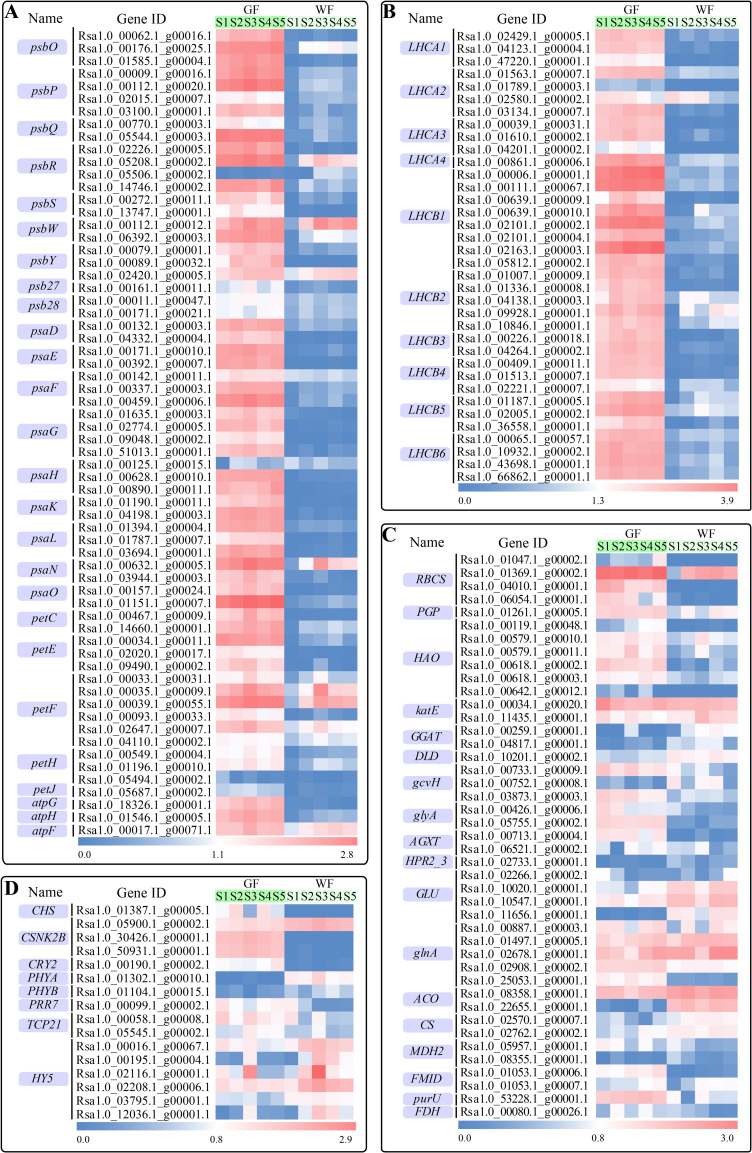
Expression profiles of DEGs involved in photosynthesis-related pathways in GF and WF flesh at five developmental stages. **A**, Photosynthesis pathway. psbO, photosystem II oxygen-evolving enhancer protein 1; psbP, photosystem II oxygen-evolving enhancer protein 2; psbQ, photosystem II oxygen-evolving enhancer protein 3; psbR, photosystem II 10kDa protein; psbS, photosystem II 22kDa protein; psbW, photosystem II PsbW protein; psbY, photosystem II PsbY protein; psb27, photosystem II Psb27 protein; psb28, photosystem II 13kDa protein; psaD, photo-system I subunit II; psaE, photosystem I subunit IV; psaF, photosystem I subunit III; psaG, photosystem I subunit V; psaH, photosystem I subunit VI; psaK, photosystem I subunit X; psaL, photosystem I subunit XI; psaN, photosystem I subunit PsaN; psaO, photosystem I subunit PsaO; petC, cytochrome b6-f complex iron-sulfur subunit; petE, plastocyanin; petF, ferredoxin; petJ, cytochrome c6; petH, ferredoxin—NADP+ reductase; atpF, F-type H+-transporting ATPase subunit b; atpH, F-type H+-transporting ATPase subunit delta; atpG, F-type H+-transporting ATPase subunit gamma. **B**, Photosynthesis–antenna proteins pathway. LHCB, light-harvesting complex II Chl a/b binding protein; LHCA, light-harvesting complex I Chl a/b binding protein. **C**, Glyoxylate and dicarboxylate metabolism pathway. PGP, phosphoglycolate phosphatase; HAO, (S)-2-hydroxy-acid oxidase; katE, catalase; GGAT, glutamate-glyoxylate amino-transferase; DLD, dihydrolipoamide dehydrogenase; gcvH, glycine cleavage system H protein; glyA, glycine hydrox-ymethyltransferase; AGXT, alanine-glyoxylate transaminase; HPR2_3, glyoxylate/hydroxypyruvate reductase; GLU, glutamate synthase (ferredoxin); glnA, glutamine synthetase; ACO, aconitate hydratase; CS, citrate synthase; FMID, formamidase; purU, formyltetrahydrofolate deformylase; FDH, formate dehydrogenase. **D**, Circadian rhythm pathway. CHS, chalcone synthase; CSNK2B, casein kinase II subunit beta; CRY2, cryptochrome 2; PHYA, phytochrome A; PHYB, phytochrome B; PRR7, pseudo-response regulator 7; TCP21, transcription factor TCP21; HY5, transcription factor HY5.

A total of 64 genes specifically involved in the photosynthesis pathway were differentially expressed between GF and WF tissues in the present study, including 23 genes associated with the PSII core, 23 genes associated with the PSI core, two genes associated with the cytochrome b6/f complex, 13 genes associated with photosynthetic electron transport and three genes associated with F-type ATPase (Fig 9A). Among these 64 genes, a vast majority of the genes (up to 75%) were significantly up-regulated at all five stages in GF tissues.

The antenna system of photosynthesis consists of proteins encoded by light-harvesting Chl a/b binding (*LHC*) genes. These *LHC* genes are divided into two sub-classes: *LHCA* associated with photosynthesis I (PS I) and *LHCB* associated with photosynthesis II (PS II). A total of 36 *LHC* genes, including 11 *LHCA* genes (three *LHCA1*, four *LHCA2*, three *LHCA3* and one *LHCA4*) and 25 *LHCB* genes (eight *LHCB1*, five *LHCB2*, two *LHCB3*, three *LHCB4*, three *LHCB5* and four *LHCB6*), were differentially expressed between GF and WF during the process of taproot development (Fig 9B). Thirty-three of the above 36 genes showed remarkably higher expression levels across different stages in GF than those in WF tissues; Rsa1.0_02580.1_g00002.1 (*LHCA2*) mRNA content in GF was significantly higher than that in WF tissues at S4, and Rsa1.0_01789.1_g00003.1 (*LHCA2*) and Rsa1.0_02221.1_g00007.1 (*LHCB4*) were significantly up-regulated at all five stages except S4.

In total, 43 genes involved in the pathway of glyoxylate and dicarboxylate metabolism showed differential expression between the WF and GF during radish growth (Fig 9C). These genes were predominantly concentrated in the photorespiration and glyoxylate cycles. For example, *RBCS* (4), *PGP* and *HAO* (6), which convert Ribulose-1,5-P2 to glyoxylate in photorespiration, had significantly higher expression levels in GF than those in WF. The expression levels of the four genes associated with the degradation of glyoxylate into NH_3_ and CO_2_, *GGAT*, *DLD* and *gcvH* (Rsa1.0_00752.1_g00008.1), were significantly reduced in GF compared with those in WF, while the other two *gcvH* genes (Rsa1.0_00733.1_g00009.1 and Rsa1.0_03873.1_g00003.1) showed the opposite trend. The five key genes involved in the conversion of glycine to D-glycerate showed complex expression patterns. Three of these, Rsa1.0_00426.1_g00006.1 (*glyA*), Rsa1.0_05755.1_g00002.1 (*glyA*), and Rsa1.0_00713.1_g00004.1 (*AGXT*), showed consistently higher expression levels in GF than those in WF across the five stages. Rsa1.0_06521.1_g00002.1 (*AGXT*) exhibited lower expression level at S2 but showed higher expression level at S4 and S5 in GF than that in WF. *HPR2_3* (Rsa1.0_02733.1_g00001.1), which can convert glyoxylate to glycolate, exhibited down-regulated expression at S1 in GF compared with that in WF, but presented no differential expression across the other four stages. Four *GLU* and five *glnA* genes involved in the interconversion between glutamine and glutamate were identified in this study. All four *GLU* and two *glnA* genes (Rsa1.0_01497.1_g00005.1 and Rsa1.0_02678.1_g00001.1) showed reduced expression levels in at least one stage in GF compared with those in WF, whereas the other three *glnA* genes (Rsa1.0_00887.1_g00003.1, Rsa1.0_02908.1_g00002.1 and Rsa1.0_25053.1_g00001.1) showed increased expression levels. KatE converts H_2_O_2_ to O_2_, and we identified two *katE* genes in the present study. These two genes exhibited different expression patterns. Rsa1.0_00034.1_g00020.1 had higher expression level in GF than that in WF, with Rsa1.0_11435.1_g00001.1 showing the opposite trend. Five genes participating in the glyoxylate cycle, including *ACO* (Rsa1.0_08358.1_g00001.1and Rsa1.0_22655.1_g00001.1), *CS* (Rsa1.0_02570.1_g00007.1 and Rsa1.0_02762.1_g00002.1) and *MDH2* (Rsa1.0_08355.1_g00001.1), showed significantly reduced expression levels in at least one stage in GF compared with those in WF. However, the expression of Rsa1.0_05957.1_g00001.1 (*MDH2*) was higher at S4 in GF than that in WF. Additionally, four genes (*FDH*, *FMID* (2), and *purU*) responsible for the conversion of N-Formylderivatices to CO_2_ were all up-regulated in GF compared with those in WF.

The circadian rhythm pathway was also significantly enriched according to the comparative transcriptome data of GF and WF. We identified 16 DEGs involved in this pathway, of which eight genes belonging to two families were transcription factors (Fig 9D), such as six *HY5* genes (bZIP transcription factors) were identified. Among them, Rsa1.0_00016.1_g00067.1, Rsa1.0_00195.1_g00004.1, Rsa1.0_02208.1_g00006.1, and Rsa1.0_12036.1_g00001.1 showed significantly lower expression levels in GF than those in WF during the stages from S2 to S5, while Rsa1.0_03795.1_g00001.1 expression was significantly lower in GF than that in WF only at S4. The expression of the other *HY5* (Rsa1.0_02116.1_g00001.1) was complicated, and was higher in GF than that in WF at S1 but lower at S2, S3, S4 and S5. One *TCP* transcription factor gene (Rsa1.0_00058.1_g00008.1) was expressed at higher level in GF than that in WF at S2 and S4, and the other (Rsa1.0_05545.1_g00002.1) only at S4. Besides the seven transcription factor genes, eight functional DEGs relevant to the circadian rhythm pathway were also identified. Of these, five genes including *PRR7*, *CRY2*, *CHS* and *CSNK2B* (Rsa1.0_30426.1_g00001.1 and Rsa1.0_50931.1_g00001.1) were expressed at higher levels in GF than those in WF. However, *PHYA*, *PHYB* and *CSNK2B* (Rsa1.0_05900.1_g00002.1) showed lower expression levels in the GF.

### Validation of candidate genes in different radish cultivars

**Fig 10:**
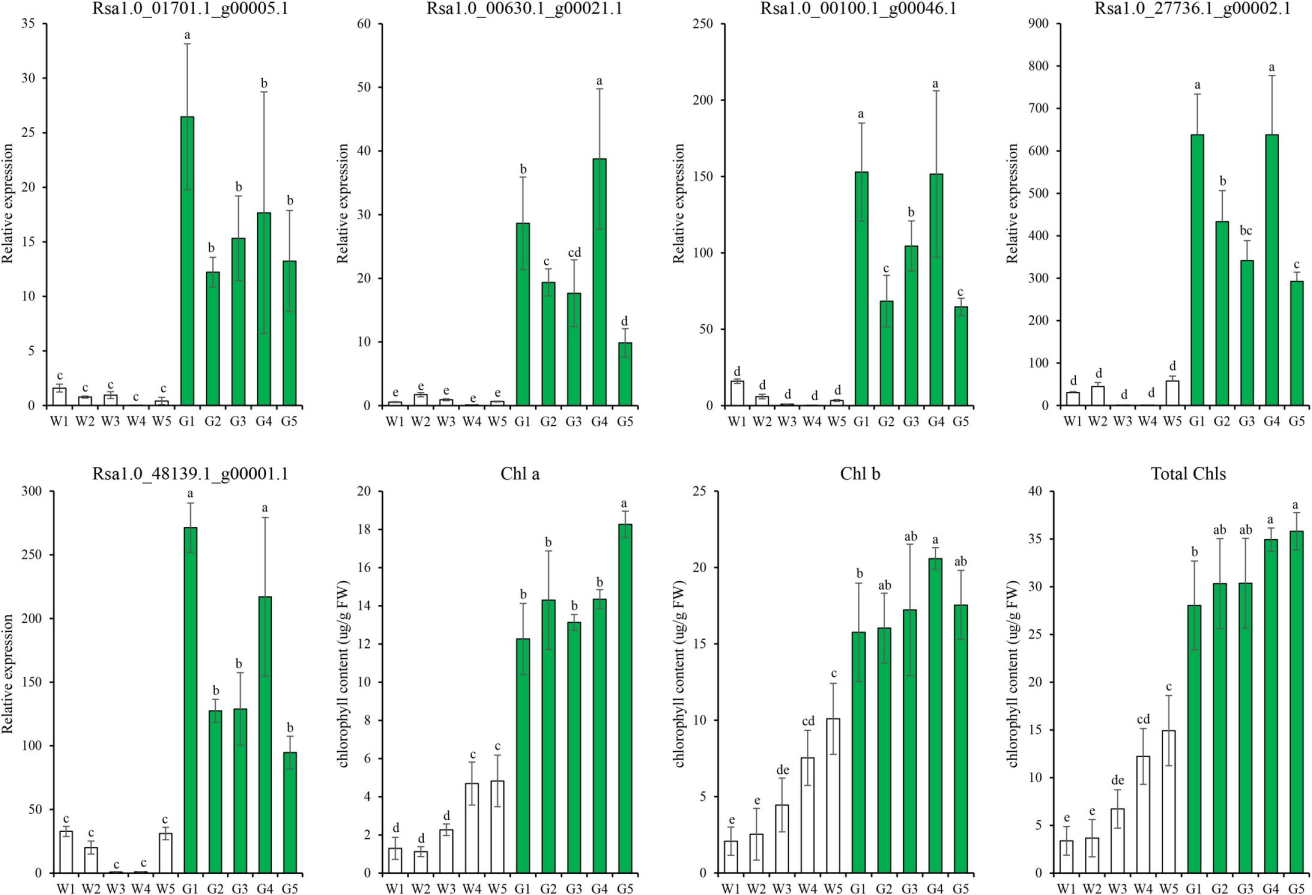
qRT-PCR expression of five candidate genes involved in part Ⅳ of chlorophyll biosynthesis and chlorophyll content in ten radish cultivars. Different letters indicate significant differences at P≤0.05. W1-W5, white-fleshed radish cultivars; G1-G5, green-fleshed radish cultivars.

Based on the correlation analysis (S3 Fig) and expression levels of DEGs (Fig 7), we selected five genes that participated in part Ⅳ of Chl biosynthesis as candidate genes for further validation in ten white- and green-fleshed cultivars (S8 Table) using qRT-PCR. These five genes were all highly expressed in green-fleshed cultivars, but showed low expression in white-fleshed cultivars (Fig 10). Therefore, our results confirmed that *chlP*, *POR*, *chlM* and *chlE* are potential candidate genes responsible for the higher Chl content in green flesh than that in white flesh samples from radish taproots.

## Discussion

To understand the molecular mechanism of green flesh formation in radish, we extracted RNA from taproots of two representative cultivars, GF (green-fleshed radish) and WF (white-fleshed radish), across five developmental stages (S1–S5). RNA sequence analysis was subsequently carried out. Furthermore, differences in gene expression levels were analyzed between GF and WF at the corresponding stages. GO annotation showed that genes related to chloroplast, chloroplast membrane, chloroplast thylakoid, Chl biosynthetic process, and photosynthetic electron transport were highly enriched among the up-regulated genes in GF. The KEGG pathway analysis also supported the results of GO enrichment, which showed that five photosynthesis-related pathways (photosynthesis, photosynthesis-antenna proteins, glyoxylate and dicarboxylate metabolism, carbon fixation, and porphyrin and Chl metabolism) were enriched at all five stages. In addition, we observed clear Chl auto-fluorescence in GF tissues. These results indicate that the green flesh of taproots may contain chloroplasts, which may be active in GF tissues.

Chl is a major photosynthetic pigment responsible for the green color of the plants. Most of the genes participating in this pathway have been identified and characterized in the leaves of higher plants [4]. Some studies have also been carried out in non-foliar tissues, such as kiwi flesh [1], tomato fruits [26], green flower petals [13], radish taproot skin [15], barley developing spikelets [27], and litchi pericarps [28]. In the current study, we found 74 genes, including 34 DEGs, involved in Chl metabolism in radish flesh (Fig 7). Pearson’s correlation analysis showed that the expression levels of approximately 80% of above 34 DEGs were significantly correlated with the Chl content (P≤0.01) (S3 Fig). However, only 12 genes, *chlI* (1), *chlM* (1), *chlE* (2), *POR* (2), *chlP* (5), and *CAO* (1), showed higher expression in GF than those in WF from S1 to S5. Interestingly, 11 of these 12 genes were involved in Part Ⅳ in Chl biosynthesis, from protoporphyrin Ⅸ to Chl a. Five *chlP* genes were identified in radish flesh, and their expression levels were consistently high in GF. *ChlP* genes encoding geranyl-geranyl reductase provide a hydrophobic alcohol moiety for Chl synthesis [29]. The *chlP* antisense tobacco plants showed a reduction in Chl content, which resulted in a pale or variegated phenotype [30], and the rice *OschlP* mutant, 502ys, accumulated lesser Chl compared with that by the wild type [29]. POR catalyzes protochlorophyllide to form the chlorophyllide. There are three POR isoforms (AtPORA, AtPORB, and AtPORC) in Arabidopsis [31]. Two POR isoforms have been identified in barley, tobacco, and rice [31–33]. However, only one *POR* gene has been detected in cucumber and pea [34,35]. Five *POR* genes with different expression patterns were detected in the transcriptome data of radish flesh. Rsa1.0_08969.1_g00001.1 and Rsa1.0_17789.1_g00001.1, orthologs of *AtPORA*, showed very low expression levels. Rsa1.0_00630.1_g00021.1 (ortholog of *AtPORB*), Rsa1.0_00161.1_g00008.1 (ortholog of *AtPORC*) and Rsa1.0_00556.1_g00016.1 (orthologs of *AtPORC*) presented higher expression in GF than that in WF flesh (S6 Table). These results indicate that three POR isoforms may exist in radish flesh, and PORB and PORC are essential for Chl formation in radish green flesh. Previous studies also found that POR was closely related to Chl content in chrysanthemum petals and peony leaves [13,36].

Genes such as *CAO*, *chlG*, *NOL*, *CLH*, and *HCAR* were expressed in the Chl cycle. CAO and chlG catalyze the formation of Chl b from chlorophyllide a and hydroxychloro-phyllide a, while NOL and CLH are responsible for the reverse progress, i.e., from Chl b to hydroxychlorophyllide a [4]. The high expression of *CAO* and *chlG* and the low expression of *NOL* and *CLH* in GF suggest that Chl b synthesis was more active than its degradation in GF. This is one of the possible reasons for the accumulation of Chl b in radish green flesh. In addition, HCAR encoding hydroxymethyl Chl a reductase was not detected in the current study. This result differed from previous studies, which showed that a high expression level of *HCAR* was detected in plant green tissues [13,15]. This difference may be due to the specific tissue (radish green flesh) used in our study. Furthermore, only three DEGs involved in Chl degradation were identified in this study, and the expression of these genes was up-regulated in GF at only one stage, which may indicate that Chl degradation was not active in GF at most stages.

In GF, the contents of Chl a, Chl b and total Chl decreased at S2 compared to those in other stages (Fig 3). A previous study on the cyanobacterium *Synechocystis* [37] reported that the amount of Chl a decreased with increasing growth rate. We found that the taproot relative growth rate of GF between S2 and S1 was 343.31%, which was much faster than that between other stages (S4 Fig). This decrease in S2 may therefore be caused by the rapid growth of GF taproots from S1 to S2. In addition, we examined the expression trends of the 34 DEGs involved in Chl metabolism in GF across five stages (especially at S2), and found that the expression levels of these genes did not significantly decrease at S2 compared to those in other stages (Fig 7). We further analyzed the correlation between the expression levels of these 34 DEGs and Chl content only in GF. Unlike in both GF and WF, no obvious positive correlation was apparent in GF alone (S9 Table). Previous studies have shown that leaf Chl content is associated with post-transcriptional regulation [38,39], which may also have an effect on the changes in Chl content at different developmental stages in GF. Future experiments are required to explore this possibility.

Leaf photosynthesis is a well-established and well-researched process [40]. However, leaves are not the only part of the plant where photosynthesis can occur. Photosynthesis also occurs in many non-foliar organs, such as in cucumber fruit, Arabidopsis silique walls, and cotton stems, bracts and capsule walls [18,41,42]. The functions of the electron transport chain have been detected even in the pith of the tree stem [17]. In our study, numerous genes associated with photosynthesis were identified, and the vast majority of these genes showed high expression levels in GF (S6 Table). For example, four *RBCS* genes were highly expressed in GF, however three of them showed low expression in WF. *RBCS* genes encoding the small subunit of RUBISCO are regarded as key markers of photosynthesis. ALDO and SBPase are two essential enzymes involved in the Calvin cycle. Previous studies have shown that over expression of *SBPase* and/or *ALDO* in transgenic plants can result in enhanced photosynthesis [43–46]. We found six *ALDO* genes and one *SBPase* gene that were differentially expressed between GF and WF. Without exception, all these genes exhibited high expression in the GF. Additionally, many key genes participating in photosynthetic electron transport (e.g., *petC*, *petH*, *psaH*, *psaK*, *atpH*, *atpG*, *Lhca*, *Lhcb*) and photorespiration (e.g., *PGP*, *HAO*, *gcvH*, *glyA*, *glnA*, *purU*) also showed high expression in GF, although the expression levels of Rsa1.0_05506.1_g00002.1 (*psbR*) and Rsa1.0_00125.1_g00015.1 (*psaH*) decreased in GF (Fig 9), probably because of their redundant functions. Chl auto-fluorescence was observed, and high values of FV/FM, ΦPSII, and ETR were also detected in the GF. These results strongly suggest that photosynthesis occurs in the green flesh of GF taproots. Furthermore, while the radish is a typical C_3_ plant [47], some DEGs were arranged in the C_4_ pathway according to the KEGG analysis (Fig 8 and S7 Table). Genes involved in the C_4_ pathway are also expressed in C_3_ plants, which may perform different functions in C_4_ and C_3_ plants [48,49]. Further, no difference was detected in the expression levels of *PEPC* and *PPDK* (key genes of the C_4_ pathway) between GF and WF tissues in our transcriptome data (Fig 8 and S7 Table). Therefore, it is unlikely that the radish green flesh could perform the C_4_ photosynthetic pathway.

In this study, the circadian rhythm pathway was enriched by KEGG pathway enrichment analysis. The circadian clock is considered a developmental manager in plants [50]. Many studies have shown that circadian rhythms are closely integrated with photosynthesis [51]. Some genes involved in circadian rhythm (such as *CRY*, *PHY*, and *HY5*) also play important roles in photosynthesis. CRY and PHY are blue-light and red/far-red-light receptors, respectively [52]. Chaves et al. [53] reported that CRY2 may serve to enhance blue-light sensing under conditions where light is restricted, although CRY2 has overlapping functions with CRY1. We observed that *CRY2* showed high expression level in GF during the entire period of taproot development, while P*HYA* and *PHYB* genes exhibited barely detectable expression levels in GF. The environmental light must pass through the outer tissue to reach the inner part, the process of which reduces the light quantity and quality [17]. Sun et al. [54] reported that the inner tissues of the stem and root were exposed to an internal light environment enriched in far-red light. However, the expression levels of *CRY* and *PHY* might suggest that the radish green flesh may use weak blue-light, rather than far-red light, to activate photosynthesis, however further studies are needed to confirm this. *HY5* is a bZIP-type transcription factor [55], and it can positively regulate Chl synthesis and chloroplast biogenesis [56,57]. Therefore, our study also focused on the expression of *HY5*. Six *HY5* genes with differential expression levels between GF and WF were identified in the current study. Compared with WF, all *HY5* genes showed down-regulated expression from S2 to S5 in GF (S6 Table). The decreased expression of *HY5* genes contradicted the increased concentration of Chl in GF. This could be caused by the highly diverse functions of *HY5* [58,59].

## Conclusions

In this study, RNA-Seq technology was used to compare the gene expression patterns between the flesh of green-fleshed GF and white-fleshed WF during the different stages of taproot development. Through a combined analysis of transcriptome data and Chl content in GF and WF, we can infer that Chl biosynthesis is likely a dynamic, regulated process in GF. Compared to that in the WF, Chl accumulation in GF may be due to the higher expression of Chl biosynthesis genes, especially those associated with Part Ⅳ, and the lower expression of Chl degradation genes, which was also supported by the expression profiling of five candidate genes in diverse cultivars. Additionally, a large number of photosynthesis-related genes were highly expressed in GF, where Chl auto-fluorescence was evident and photosynthetic activity was also observed. These results strongly suggest that the green flesh of GF can perform photosynthesis. Further studies are needed to identify the key regulated genes involved in Chl biosynthesis and to clarify how photosynthesis is initiated and then progresses in the green flesh of radish taproot.

## Supporting information

S1 Fig Five sampling stages (S1~S5) of radish taproots. (DOCX)

S2 Fig The top ten GO terms enriched in constantly up-regulated DEGs (A) and down-regulated DEGs (B) during the radish developmental stages.
(DOCX)

S3 Fig Correlation coefficients between expression levels of 34 DEGs involved in Chl metabolism and contents of Chl a, Chl b, and total Chl for each stage. (DOCX)

S4 Fig Taproot relative growth rate of GF during different developmental stages. (DOCX)

S1 Table List of primers used for qRT-PCR in this study. (XLSX)

S2 Table The data of qRT-PCR and RNA-Seq for Fig 4. (XLSX)

S3 Table The expression profiles of total DEGs. (XLSX)

S4 Table GO enrichment analyses of DEGs. (XLSX)

S5 Table The enriched KEGG pathways. (XLSX)

S6 Table DEGs involved in the ten enriched pathways. (XLSX)

S7 Table Genes involved in carbon fixation pathway. (XLSX)

S8 Table The radishes used for validation by qRT-PCR. (XLSX)

S9 Table Correlation coefficients between expression levels of 34 DEGs involved in Chl metabolism and contents of Chl a, Chl b and total Chl only in GF. (XLSX)

S10 Table The raw data of photochemical efficiency and ETR for Fig 2. (XLSX)

S11 Table The raw data of Chl content for Fig 3. (XLSX)

S12 Table The expression data of all genes. (XLSX)
