# Supplementary material for: Comparative transcriptome analysis identifies genes associated with chlorophyll levels and reveals photosynthesis in green flesh of radish taproot
Source: PLoS One. 2021 May 27;16(5):e0252031. doi: 10.1371/journal.pone.0252031 (PMC8158985; doi:10.1371/journal.pone.0252031)
Supplement: S1 Fig — (DOCX) [file pone.0252031.s001.docx]

WF

S1 (September 25th)


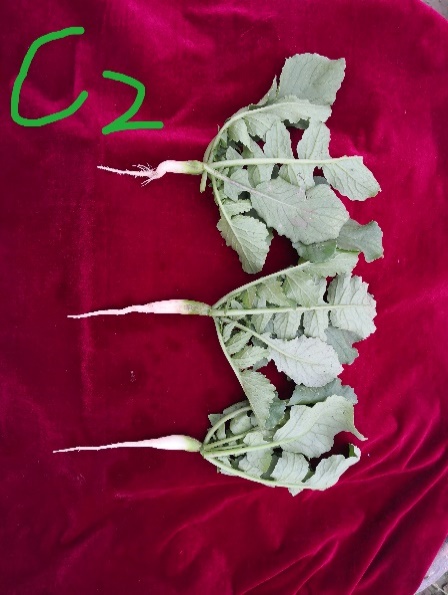


GF

S2 (October 2nd)


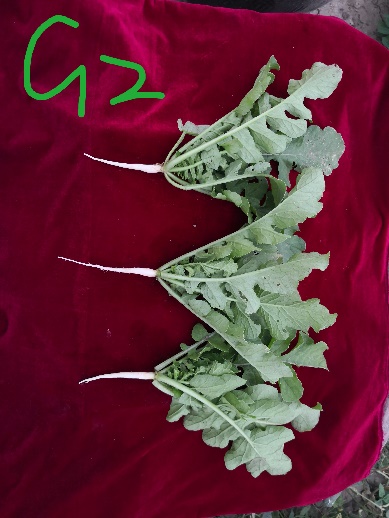

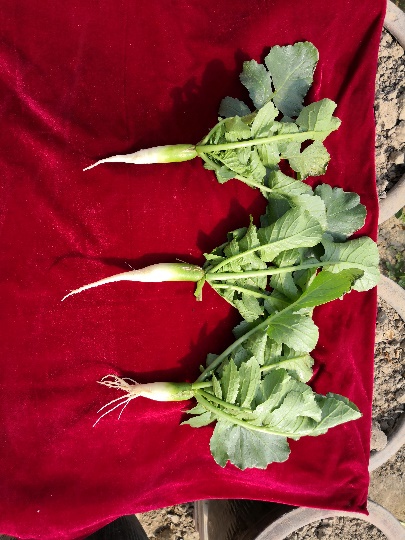

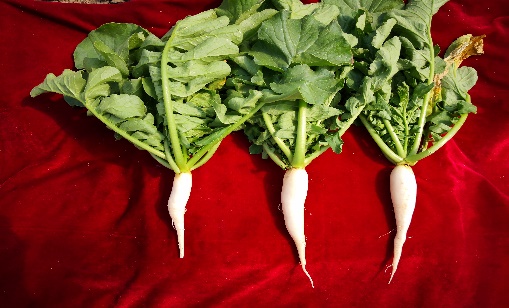


S3 (October 9th)


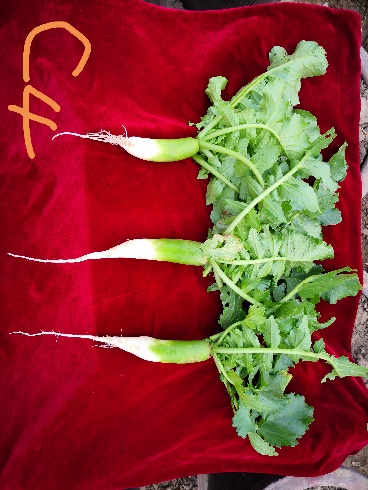

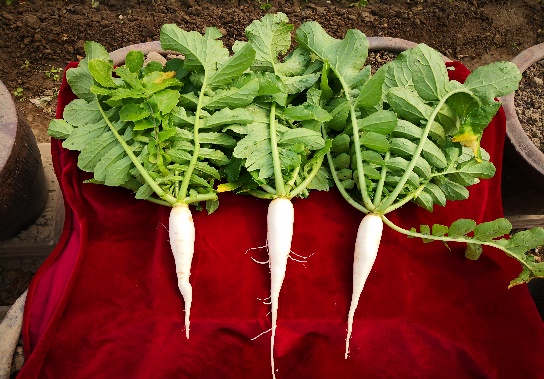


S4 (October 16th)


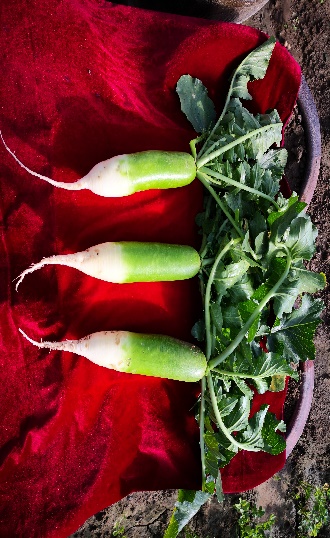

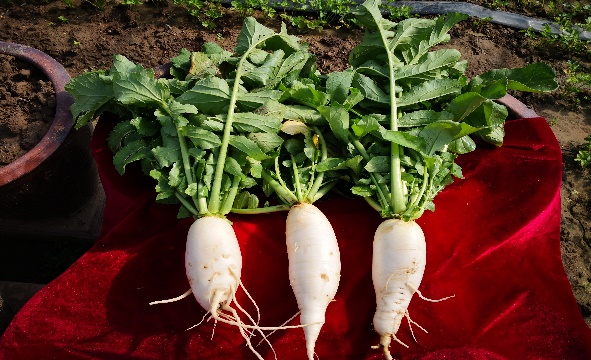


S5 (October 23th)


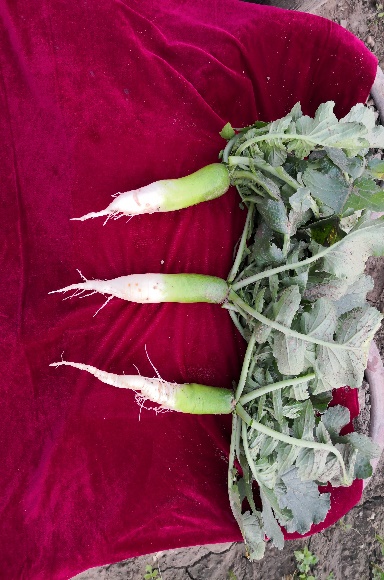

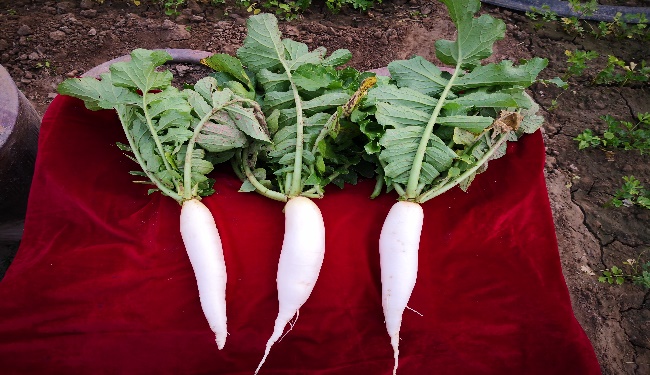


**Fig. S1** Five sampling stages (S1~S5) of radish taproots. GF, Cuishai. WF, Zhedachang. Blue boxes indicate the sampling locations.
