# Supplementary material for: Comparative transcriptome analysis identifies genes associated with chlorophyll levels and reveals photosynthesis in green flesh of radish taproot
Source: PLoS One. 2021 May 27;16(5):e0252031. doi: 10.1371/journal.pone.0252031 (PMC8158985; doi:10.1371/journal.pone.0252031)
Supplement: S2 Fig — The top ten GO terms enriched in constantly up-regulated DEGs (A) and down-regulated DEGs (B) during the radish developmental stages. (DOCX) [file pone.0252031.s002.docx]

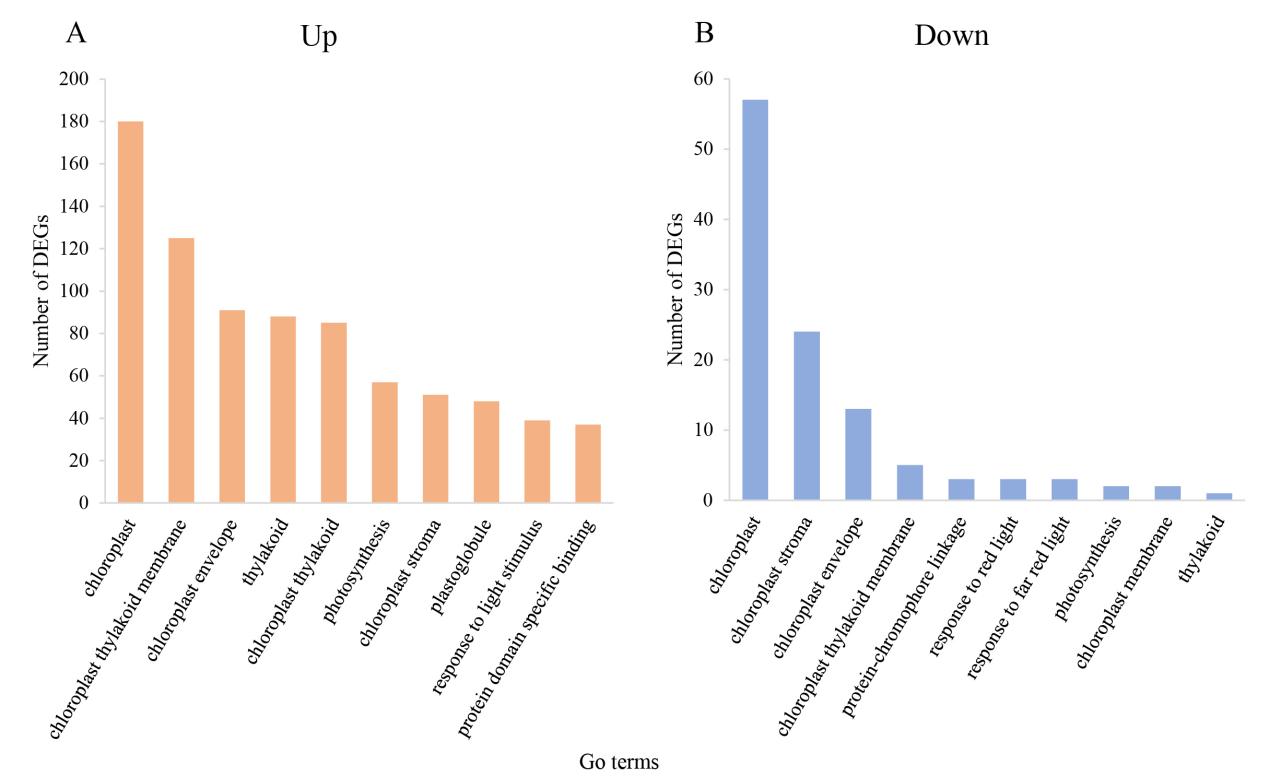


**Fig. S2** The top ten GO terms enriched in constantly up-regulated DEGs (**A**) and down-regulated DEGs (**B**) during the radish developmental stages.
