# Supplementary material for: Comparative transcriptome analysis identifies genes associated with chlorophyll levels and reveals photosynthesis in green flesh of radish taproot
Source: PLoS One. 2021 May 27;16(5):e0252031. doi: 10.1371/journal.pone.0252031 (PMC8158985; doi:10.1371/journal.pone.0252031)
Supplement: S3 Fig — (DOCX) [file pone.0252031.s003.docx]

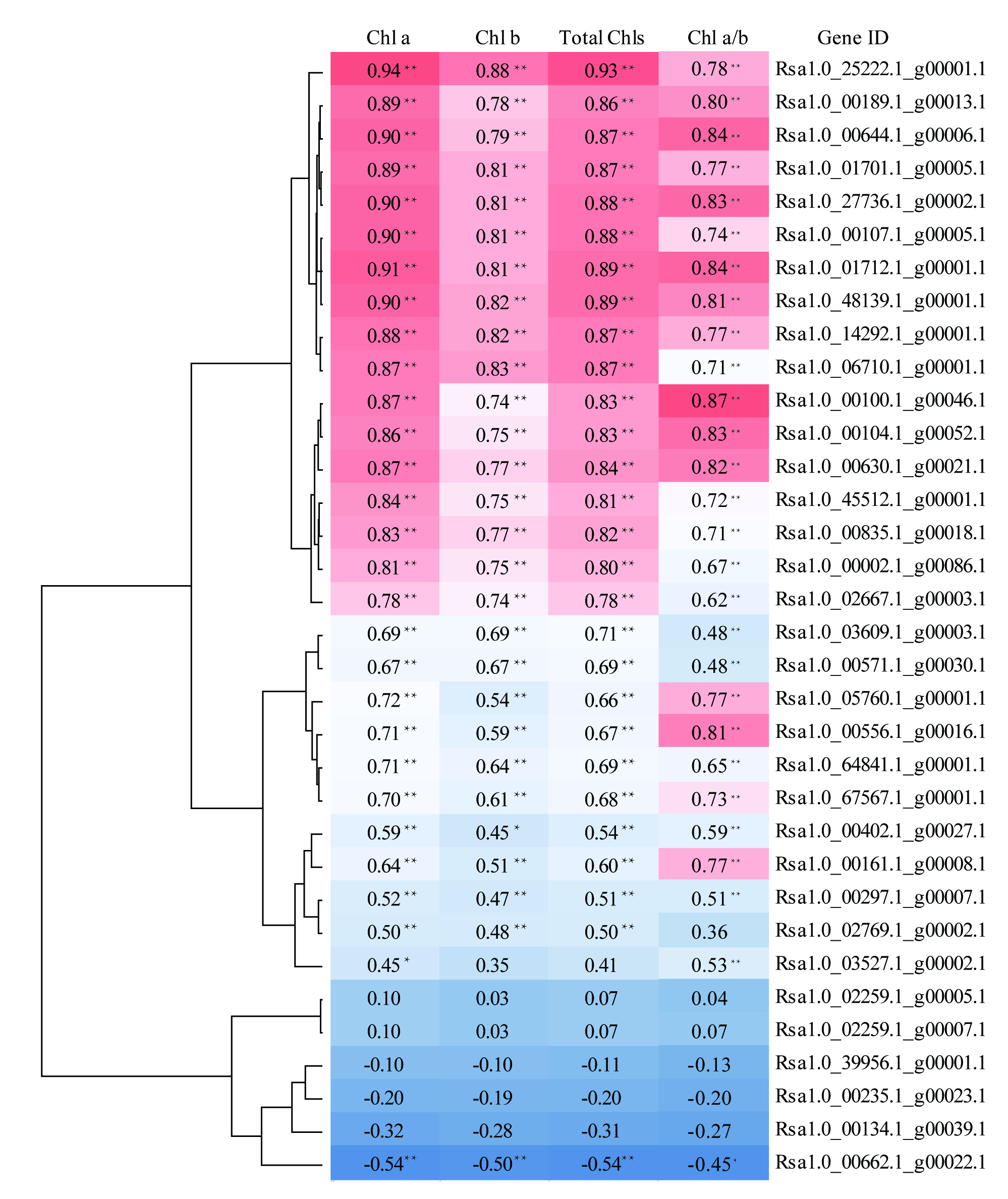


**Fig. S3** Correlation coefficients between expression levels of 34 DEGs involved in Chl metabolism and contents of Chl a, Chl b, total Chls, and Chl a/b for each stage. *Significant difference at P≤0.05, **Significant difference at P≤0.01.
