# Supplementary material for: Comparative transcriptome analysis identifies genes associated with chlorophyll levels and reveals photosynthesis in green flesh of radish taproot
Source: PLoS One. 2021 May 27;16(5):e0252031. doi: 10.1371/journal.pone.0252031 (PMC8158985; doi:10.1371/journal.pone.0252031)
Supplement: S4 Fig — (DOCX) [file pone.0252031.s004.docx]

**Fig. S4** Taproot relative growth rate of GF during different developmental stages. Taproot relative growth rate was calculated according to the formula: Taproot relative growth rate (%) = (W_Sn+1_-W_Sn_)/W_Sn_*100, where W_Sn+1_ and W_Sn_ are the weight of taproot at stage Sn+1 and Sn (n=1~4), respectively.
